# Effect of Fluticasone and Salmeterol on Tracheal Responsiveness to Ovalbumin and Lung Inflammation, Administrated during and after Sensitization

**DOI:** 10.1155/2014/865292

**Published:** 2014-01-19

**Authors:** Zahra Gholamnezhad, Mohammad Hossain Boskabady, Mohammad Reza Khazdair, Mahmoud Hosseini, Mahdi Abbasnejad

**Affiliations:** ^1^Neurogenic Inflammation Research Centre, School of Medicine, Mashhad University of Medical Sciences, Mashhad 9177948564, Iran; ^2^Department of Biology, Faculty of Sciences, Kerman University of Shahid Bahonar, Kerman 9177948564, Iran

## Abstract

The effect of duration of administration of fluticasone propionate and salmeterol on tracheal responsiveness to ovalbumin and total and differential white blood cell in sensitized guinea pig was examined. Six groups of guinea pigs (*n* = 7) were sensitized to ovalbumin. Three groups of them were subjected to inhaled fluticasone propionate and salmeterol, one group during sensitization (A), one group after that (for 18 days, B), and the other one during sensitization but with 18 days delay before measurements (C). Three other groups were treated with placebo in the same manner. The tracheal responsiveness to ovalbumin and total and differential white blood cells of three placebo groups were significantly higher than those of control group (*P* < 0.001 for all cases). Tracheal responsiveness to ovalbumin and total and differential white blood cell in treated groups with fluticasone propionate and salmeterol were significantly decreased compared to those of placebo groups (nonsignificant to *P* < 0.001). The improvement in all variables in treatment groups A and C were more pronounced than group B. The results showed that fluticasone propionate and salmeterol had a prevention effect on tracheal hyperresponsiveness to ovalbumin and lung inflammation which was more pronounced when administered during than after sensitization.

## 1. Introduction

The most important characteristic feature of asthma is a chronic inflammatory disorder of the airway [1] which leads to airway hyperresponsiveness (AHR) [2]. Asthma is a two-component disease including airway inflammation [3] and smooth muscle dysfunction [4]. Therefore, the most effective treatment is a drug which targets both components of the disease.

The combination of long acting *β*-agonists (LABA) and inhaled corticosteroid is more effective in treatment of asthma than increasing the dose of inhaled corticosteroid (ICS) [5–8]. Suppression of the inflammatory process by LABA is also indicated in *in vitro *and *in vivo *animal studies [9–11]. Treatment with fluticasone propionate (FP) and salmeterol (SM) improved allergen-induced airway remodeling [12] and was able to control peripheral blood T-cell activation in asthmatic patients more efficiently [12]. Bidirectional interaction between *β*-agonists and steroids on various pathophysiologic aspects of asthma was documented previously [13, 14].

It was well documented that regular treatment with FP and SM combination resulted in continuous improvement in AHR with maintenance of asthma control in the majority of patients [15–17]. However, there are still many questions to be answered in this field. Most studies examined the effect of FP and SM combination on lung inflammation and airway responsiveness administered after clinical manifestation of asthma both in human [15–17] and in animal models [18] and only in few studies the effect of combined therapy was examined during sensitization in animal [19].

However, the effect of combined therapy, administered during, or after sensitization on the asthmatic airway inflammation is not compared yet. Therefore, in this study, the effect of an inhaled corticosteroid, fluticasone propionate and long acting *β*-agonist, salmetrol during and after sensitization of guinea pigs was investigated in tracheal responsiveness to ovalbumin (OA) and total and differential white blood cell (WBC) in bronchoalveolar lavage. In addition, the effect of an allergen-free period on the efficacy of combined therapy was also examined.

## 2. Materials and Methods

### 2.1. Animal Sensitization and Animal Groups

Guinea pigs were sensitized to (OA) as previously described [20–22]. Briefly, 10 mg OA (Sigma Chemical Ltd, UK) and 100 mg Al(OH)_3_ dissolved in 1 mL saline were administered via i.p injection on day one and seven. The animals were exposed to an aerosolized OA solution 4% for 18 ± 1 days, each day 4 min from day 17.

The aerosol was administered in a closed chamber, dimensions 30 × 20 × 20 cm. The study was approved by the ethical committee of the Mashhad University of Medical Sciences.

### 2.2. Animal Groups

**Figure 1:**
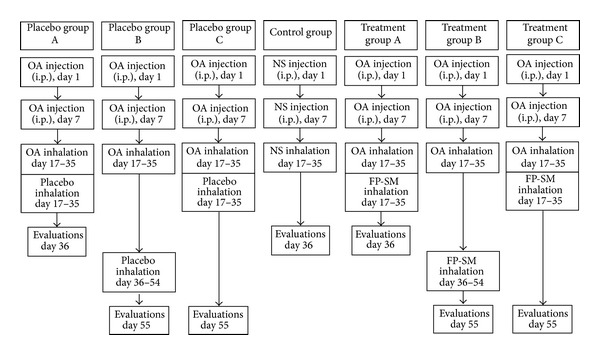
Description of control, three placebo, and three treated sensitized groups. FP: fluticasone propionate (250 *μ*g); SM: salmeterol (100 *μ*g); OA: ovalbumin; NS: normal saline.

Guinea pigs were randomly divided into seven groups (*n* = 7 for each group) as follows (Figure 1).

- Control group (group C): receiving Al(OH)_3_ alone dissolved in 1 mL normal saline and inhaled saline aerosol instead of OA.
- Treatment and placebo groups A: treated with 250 *μ*g inhaled FP twice/day + 100 *μ*g inhaled SM twice/day or placebo (composition; CFC-free propellant HFA as4a; 1,1,1,2-Tetra fluoro ethane) (GlaxoSmithKline Research Triangle, NC) during sensitization period for 18 days.
- Treatment and placebo groups B: treated with FP + SM or placebo after sensitization period for 18 days.
- Treatment and placebo groups C: treated with FP + SM or placebo during sensitization period and evaluated with 18 days delay.

Aerosol FP and placebo were administered using ordinary canister through a modified spacer as previously described [23].

## 3. Experimental Design

### 3.1. Tissue Preparations

Guinea pigs were sacrificed and their trachea was removed. Each trachea was cut into 10 rings (each containing 2-3 cartilaginous rings). All the rings were then cut open opposite the tracheal muscle and sutured together to form a tracheal chain [24].

The tissue was then suspended in a 10 mL organ bath (Schuler organ bath type 809, March-Hugstetten, Germany) containing Krebs-Henseliet solution of the following composition (mM): NaCl 120, NaHCO_3_ 25, MgSO_4_ 0.5, KH_2_PO_4_ 1.2, KCl 4.72, CaCl_2_ 2.5, and dextrose 11. The Krebs solution was maintained at 37°C and gassed with 95% O_2_ and 5% CO_2_. The tissue was suspended under an isotonic tension of 1 g and allowed to equilibrate for at least 1 h, while it was washed with Krebs solution every 15 mins.

Responses were measured using the Vernier control-type 850 N sensor with sensitivity range of 0–20 g and resolution of 0.2 mm/turn (Hugo-Sachs Elektronik, Germany) and amplified by amplifier (ML/118 quadribridge amp, March- Hugstetten, Germany) and recorded by powerlab (ML-750, 4 channel recorder, March- Hugstetten, Germany).

### 3.2. Measurement of Tracheal Response to Ovalbumin

The tracheal response to 0.1% solution of OA was measured as follows: 0.25 mL of 4% OA solution was added to the 10 mL organ bath. The degree of tracheal chain contraction was recorded after 2.5 mins and was expressed as proportion (percentage) to contraction obtained by 10 *μ*M methacholine.

### 3.3. Lung Lavage and Its White Blood Cells Count

The lungs were lavaged with 2 mL of saline for 5 times (total: 10 mL). One mL of bronchoalveolar lavage (BAL) fluid was stained with Turk solution (1 mL of Glacial Acetic Acid, 1 mL of Gentian Violet Solution 1% and 100 mL Distilled Water) and total WBC was counted in duplicate in a hemocytometer (in a Burker chamber).

The remaining BAL was centrifuged at 2500 ×g at 4°C for 10 min. The supernatant was removed. The smear was prepared from the cells and stained with Wright-Giemsa. Differential cell analysis was carried out under a light microscope by counting 400 cells and the percentage was calculated.

### 3.4. Statistical Analysis

The percent improvement in each treatment group was calculated; in cases, the treatment group's data was greater than that of corresponding placebo as calculated by ([(Treatment_A1_ − Placebo_A1_)/Placebo_A1_] × 100); in cases, the treatment group's data were lower than that of corresponding placebo, and the improvement was calculated by ([(Placebo_A1_ − Treatment_A1_)/Treatment_A1_] × 100).

All data were quoted as mean ± SEM. Percent improvements were achieved as follows: in cases, the treatment data was greater than that of corresponding placebo, the data obtained in treatment group minus the data obtained in corresponding placebo group was divided by the data obtained in the same placebo group and multiplied by 100 (e.g., [(Treatment_A1_ − Placebo_A1_)/Placebo_A1_] × 100). In cases, the treatment data was lower than that of corresponding placebo; the data obtained in placebo group minus the data obtained in corresponding treatment group was divided by the data obtained in the same treatment group and multiplied by 100 (e.g., [(Placebo_A1_ − Treatment_A1_)/Treatment_A1_] × 100). The data of three placebo groups were compared with the data of treated guinea pigs using unpaired *t*-test. The comparison of data between three treatment groups, three placebo groups and six groups of animals treated with the FP and SM and placebo with control animals, was done using one-way analysis of variance (ANOVA) with Tukey-Kramer posttest. Significance was accepted at *P* < 0.05. All statistical analyses was performed using Instat software version 3.00 (GraphPad Software, San Diego, California, USA).

## 4. Results

### 4.1. Tracheal Response to Ovalbumin

**Figure 2:**
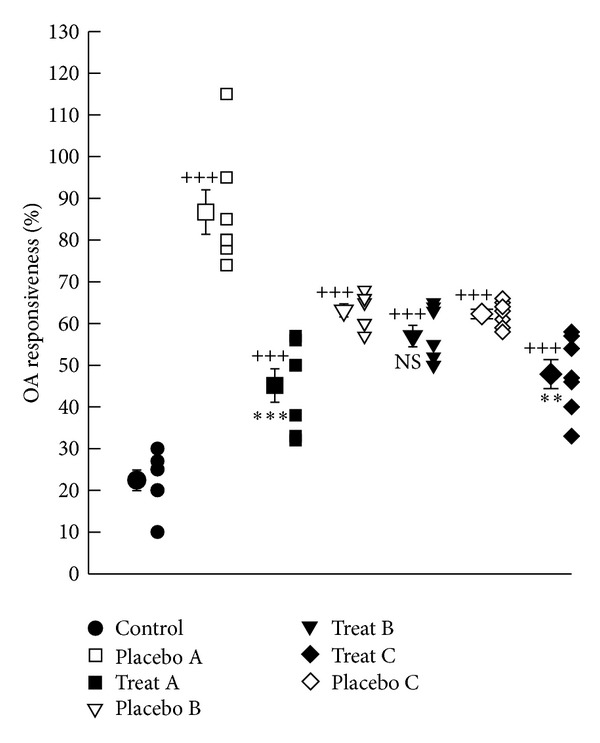
Individual values and mean ± SEM (big symbols with bars) of tracheal response to ovalbumin (OA) in control group and sensitized groups treated with fluticasone + salmeterol and placebo during (A), after (B), and during sensitization period and 18 days delay (C). Tracheal responsiveness to OA was measured by percent contraction obtained by 0.1% solution of OA compared to 10 *μ*M methacholine. Comparisons of the data between control and three treated and three placebo groups were done using one-way analysis of variance (ANOVA) with Tukey-Kramer posttest, ^++^: *P* < 0.01, and ^+++^: *P* < 0.001. Comparison of the data between each treated group with fluticasone propionate + salmeterol and corresponding placebo group was done using unpaired *t*-test, **: *P* < 0.01, ***: *P* < 0.001, NS: nonsignificant.

Tracheal responses to OA in all placebo groups (A, B, and C) were significantly higher than control group (*P* < 0.001 for all cases), (Figure 2). Tracheal responses to OA in treatment groups A and C were significantly lower than corresponding placebo groups (*P* < 0.001 and *P* < 0.01 resp.). Improvement of tracheal responsiveness to OA in treatment groups B and C was significantly lower than that of group A (*P* < 0.01 and *P* < 0.05, resp.), (Table 1).

### 4.2. Total and Differential White Blood Cell Count

**Figure 3:**
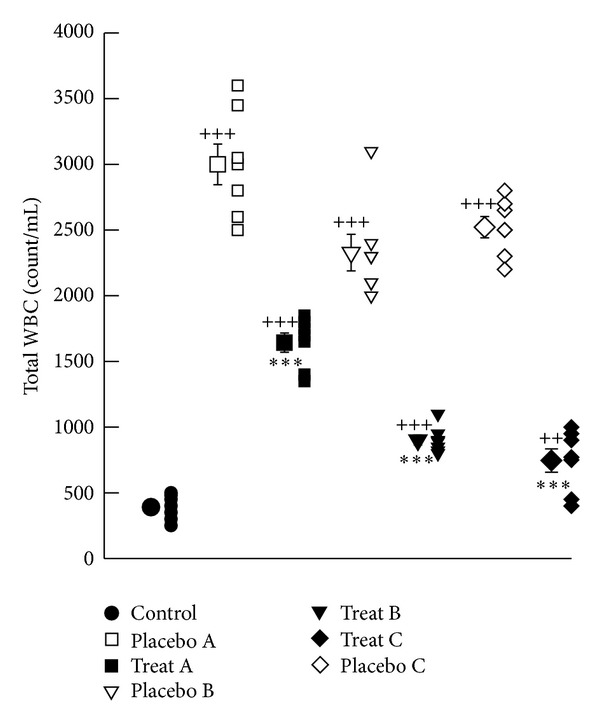
Individual values and mean ± SEM (big symbols with bars) of total WBC count (count/mL) (b) in control group and sensitized groups treated with fluticasone + salmeterol and placebo during (A), after (B) and during sensitization period and 18 days delay (C). Tracheal responsiveness to OA was measured by percent contraction obtained by 0.1% solution of OA compared to 10 *μ*M methacholine. Comparison of the data between control and three treated and three placebo groups were done using one way analysis of variance (ANOVA) with Tukey-Kramer posttest, ^++^: *P* < 0.01, and ^+++^: *P* < 0.001. Comparison of the data between each treated group with fluticasone propionate + salmeterol and corresponding placebo group was done using unpaired *t* test, **: *P* < 0.01, ***: *P* < 0.001, NS: nonsignificant.

**Figure 4:**
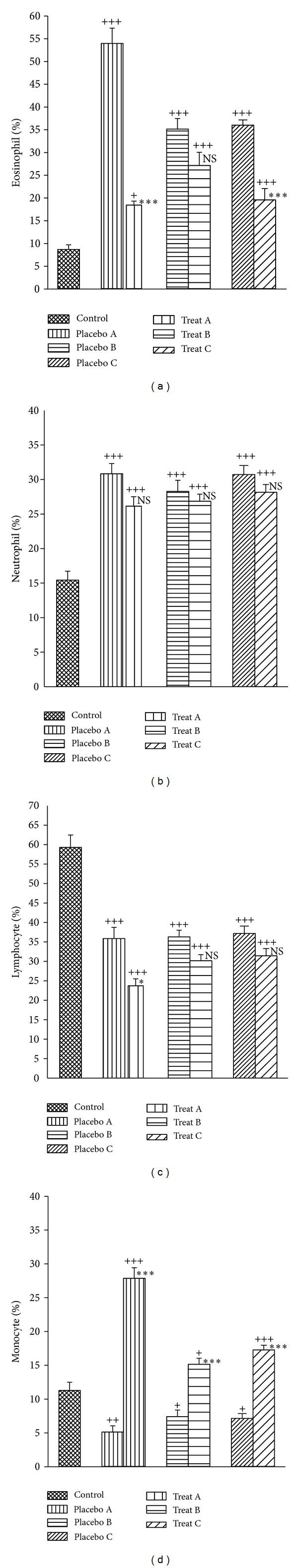
Mean ± SEM of the percentages of eosinophils (a), neutrophils (b), lymphocytes (c), and monocytes (d) of lung lavage in control group and sensitized groups treated with fluticasone + salmeterol and placebo during (A), after (B), and during sensitization period and 18 days delay (C). Comparison of the data between control and three treated with fluticasone propionate + salmeterol and three placebo groups was done using one-way analysis of variance (ANOVA) with Tukey-Kramer posttest. ^+^: *P* < 0.05, ^++^: *P* < 0.01, and ^+++^: *P* < 0.001. Comparison of the data between each treated group with fluticasone propionate + salmeterol and corresponding placebo group was done using unpaired *t*-test, NS: nonsignificant, *: *P* < 0.05, ***: *P* < 0.001, and NS: nonsignificant.

The mean values of total white blood cell (WBC) as well as the percentage of neutrophils and eosinophils were significantly higher but percentage of lymphocytes and monocytes in BAL of placebo groups A, B and C were significantly lower than those of control group (*P* < 0.01 to *P* < 0.001), (Figures 3 and 4(a)–4(d)). The total number of WBCs in treatment groups A, B, and C showed significant improvement compared to that of corresponding placebo groups (*P* < 0.001 for all cases), (Figure 3). Improvement of change in total white blood cell (WBC) in BAL in treatment group A was significantly lower than that of treatment group C (233.28 ± 33.99, *P* < 0.05), (Table 1).

There were a significant increase in the percentage of monocytes in all treatment groups and a significant decrease in the percentage of eosinophils and further decrease of lymphocytes in treatment group A compared to the corresponding placebo groups (*P* < 0.05 to *P* < 0.001), (Figure 4(d)).

The mean values of improvement in percentage of eosinophils, lymphocytes, and monocytes in BAL of treatment group A were significantly greater than those of treatment protocol B (*P* < 0.01 to *P* < 0.001). Improvement in the percentage of lymphocytes and monocytes in group A was also significantly greater than that of treatment group C (*P* < 0.01 and *P* < 0.05 resp.), (Table 1). In addition, the improvement in the percentage of eosinophils in BAL of treatment group C was significantly greater than that of treatment group B (*P* < 0.01), (Table 1).

## 5. Discussion

Tracheal response to OA, total WBC count in lung lavage and percentage of eosinophils and neutrophils increased but percentages of, lymphocytes and monocytes decreased in sensitized compared to control animals which were similar to the results of the previous studies [22, 25, 26]. The reduction in lymphocyte seen in this study may arise from the increase in total WBC number [27, 28].

The most effective agent in asthma therapy is a drug which targets both airway inflammation and smooth muscle dysfunction of the disease. Therefore, the effect of FP and SM on the tracheal responsiveness to OA, total differential WBC count which was administered during and after sensitization and during sensitization with 18 days delay in measurements was examined in this study.

The small effect of FP and SM on percentages of neutrophils in BAL fluid of sensitized animals observed in the present study may suggest that fluticasone propionate prolongs human neutrophil survival by inhibiting apoptosis by having an effect on glucocorticoid receptor [29]. Reduction in the percentages of lymphocytes in BAL of all treatment groups could be due to apoptotic effect of FP and SM which was seen in cultured lymphocytes too [30]. The increase in the percentage of monocytes in all treatment groups seen in the present study was different from the previous observed effect of corticosteroids, that is, reducing recruitment of monocytes to the airways [31]. But it could be due to reduction of total WBC count.

The effect of FP and SM on the tracheal responsiveness to OA and total and differential WBC count in lung lavage of sensitized guinea pigs seen in the present study, was supported by those who indicated that adding LABA leads to greater improvement in different parameters of asthma than increasing the dose of inhaled corticosteroid (ICS) [5, 8, 9, 11–14, 30]. However, the effect of FP and SM administered during (with measuring parameters immediately and after 18 days delay) and after sensitization of guinea pigs was evaluated which was the novelty of the present study.

In placebo group A (sensitized animals treated with inhaled placebo during sensitization, PA), most parameters were greater than those of placebo group B (sensitized animals treated with inhaled placebo after sensitization, PB) and placebo group C (sensitized animals treated with inhaled placebo during sensitization but measurement of different parameters were performed 18 days after the end of sensitization period, PC). There was greater sensitization in PA compared to PB and PC. The cause of these findings is perhaps due to an allergen-free period which is supported by the effect of allergen prevention in control of asthma disease [32] and allergen-free period in an animal study [33].

The novelty of the present study is the evaluation of the effect of FP and SM administration after (examining parameters immediately or with 18 days delay) and during sensitization of guinea pigs. The effect of an allergen free period in animals treated during sensitization on the results of combined therapy was also examined. The improvement in most parameters in treatment group A and C (TA and TC) was greater than group B (TB). The cause of these findings is perhaps due to administration of FP and SM during sensitization which indicates the importance of the early as possible asthma therapy. In fact a permanent loss of pulmonary function or even induced remission in some patients in early treatment of airway inflammation is observed previously [28] which supports these findings. The cause of less improvement in total WBC in TA group compared to TB is perhaps due to the greater change in this group. There was less improvement in some parameters in group TC compared to TA. The cause of these results is perhaps a nontreated period of 18 days in this group which emphasizes the role of ongoing treatment in asthmatic patients. The results also showed incomplete prevention of tracheal responsiveness to OA and total and differential WBC in treated sensitized guinea pigs with FP and SM either during or after sensitization which are other important findings of the present study.

In a previous study, the effect of FP alone administered during or after sensitization was examined [34]. The present study aimed to investigate the following: (1) is the combination of fluticasone propionate and salmeterol different from fluticasone propionate alone? and (2) does an allergen-free period in animals treated during sensitization affect the results? Comparison of the results of two studies showed that treatment of sensitized guinea pigs with combination of FP and SM during sensitization leads to more improvement in most variables except neutrophil, count compared to animals treated with only FP. In sensitized animals treated with combination of the two drugs after sensitization, also improvement in total WBC was greater than those treated with FP. In addition, there was no significant difference in eosinophil and monocyte counts between two drugs treated after sensitization. However, improvement in tracheal responsiveness to OA, neutrophil and lymphocyte counts was greater in treated animals with FP alone than combination of FP and SM after sensitization. Therefore, the results of both studies showed that treatment during sensitization is more effective than after sensitization. In addition, the results showed that treatment of sensitized animals with FP and SM is more effective than FP alone especially in the group treated during sensitization.

While asthma is an airway inflammation disorder [1], in further studies, the effect of FP and SM administered during or after sensitization on serum and lung lavage levels of inflammatory mediators should be examined. In the present study, tracheal responsiveness was measured, but, in asthma, disorder of mostly small and medium airways is present. This limitation should be addressed in further studies by *in vivo* measurement of airway resistance in sensitized animals treated with FP and SM during or after sensitization. In addition, all aspects of asthma are not produced in sensitized animals. Therefore, related clinical studies are needed to fully address the effect of administration time of drugs on the management of asthma.

## 6. Conclusion

The results showed that FP and SM had a preventive effect on the tracheal hyperresponsiveness to OA and lung inflammation. This preventive effect was greater when drugs were administered during sensitization. The results indicate that asthma therapy should be started as soon as possible early during the development of airway inflammation in asthmatic patients. The role of an allergen-free environment in the treatment of asthma is also suggested.

## Figures and Tables

**Table 1:** Percent improvements in tracheal responses to OA and total and differential count of white blood cells in lung lavage changes in three treatment groups A, B, and C.

Parameters	Treatment group A	Treatment group B	Treatment group C
OVA	102.71 ± 23.86	11.14 ± 3.09^++^	35.14 ± 12.73*
Total WBC	109.42 ± 29.15	194 ± 20.03	233.28 ± 33.99*
Eosinophil %	204.85 ± 17.04	44.71 ± 17.85^+++^	101.42 ± 26.13^¶¶^
Neutrophil %	22.42 ± 4.20	12.14 ± 4.96	15.71 ± 3.93
Monocyte %	495.42 ± 127.90	120.42 ± 31.25^++^	155.71 ± 25.96*
Lymphocyte %	59.28 ± 9.31	20.71 ± 3.22^++^	20.71 ± 9.33**

Values are quoted as mean ± SEM. Percent improvements were achieved as follows. In cases, the treatment data was greater than that of corresponding placebo; the data obtained in treatment group minus the data obtained in corresponding placebo group was divided by the data obtained in the same placebo group and multiplied by 100 (e.g. [(Treatment_A1_ − Placebo_A1_)/Placebo_A1_]100). In cases, the treatment data was lower than that of corresponding placebo; the data obtained in placebo group minus the data obtained in corresponding treatment group was divided by the data obtained in the same treatment group and multiplied by 100 (e.g. [(Placebo_A1_ − Treatment_A1_)/Treatment_A1_]100). Comparisons of the data between three treatment groups were done using one-way analysis of variance (ANOVA) with Tukey-Kramer posttest.

Statistical significance for the difference between the data of group A versus group B: ^++^ *P* < 0.01, ^+++^ *P* < 0.001.

Statistical significance for the difference between the data of group A versus group C: *P < 0.5, **P < 0.01.

Statistical significance for the difference between the data of group B versus group C: ^¶¶^ *P* < 0.01.
